# Early penile-only metastasis of urothelial bladder carcinoma

**DOI:** 10.2144/fsoa-2020-0047

**Published:** 2020-08-24

**Authors:** Georges Abi Tayeh, Albert Semaan, Julien Sarkis, Josselin Abi Chebel, Chadi Waked

**Affiliations:** 1 Department of Urology, Faculty of Medicine, Saint-Joseph University, Beirut, Lebanon

**Keywords:** atypical metastasis, bladder neoplasm, bladder neoplasms recurrence, locally advanced bladder neoplasms, metastatic bladder carcinoma, penile metastasis, penile neoplasms, priapism, urinary bladder neoplasms, urothelial carcinoma

## Abstract

Penile metastasis rarely occurs as a unique and early distant recurrence of urothelial bladder carcinoma. A 77-year-old male underwent a radical cystoprostatecomy for a pT3a urothelial bladder cancer. Preoperative imaging workup concluded to a disease confined to the bladder. The patient consulted 5 months later for a penile induration. Computed tomography imaging revealed a suspicious penile nodule with no other nodal or visceral lesion. Total penectomy after a confirmatory biopsy confirmed the infiltration of the corpora cavernosa, the corpus spongiosum and the urethra by a urothelial metastasis of bladder cancer. Distant recurrences after radical surgery for locally advanced bladder cancer may occur as a unique early metastasis located to the penis.

## Lay abstract

We hereby present the case of a 77-year-old male who presented with a recurrence of his bladder carcinoma located solely to the penis. This was confirmed by dedicated imaging. The patient had undergone a radical cystoprostatectomy for an invasive bladder tumor 5 months earlier. The imaging workup performed preoperatively concluded that the tumor was confined to the bladder. The definitive pathological report concluded then to a complete resection of the bladder tumor.

## Case presentation

We herein report the case of a 77-year-old male, with a remote history of smoking who underwent open radical cystoprostatectomy with bilateral extended pelvic lymph node dissection with an ileal conduit urinary diversion (Bricker) for a high-grade muscle-invasive urothelial bladder carcinoma (pT2HG). The patient was diagnosed after a transurethral resection of bladder tumor performed 3 months before his radical surgery; concomitant urethroscopy showed no suspicious inflammatory or neoplastic lesions. Preoperative thoracic, abdominal and pelvic computed tomography (CT) showed no signs of suspicious nodal involvement nor distant metastatic spread. Abdominal and pelvic magnetic resonance imaging (MRI) highlighted no stigmata of extravesical extension of tumor. The patient declined neoadjuvant chemotherapy and was therefore programmed for radical cystoprostatectomy.

On definitive pathology, the patient was found to have a poorly differentiated, high grade, urothelial carcinoma (UC) infiltrating the bulk of the muscularis propria and extending to the perivesical fat. Foci of carcinoma *in situ* and lymphovascular invasion were present. No pathologic lymph node invasion was demonstrated, compatible with a tumor node metastasis (TNM) classification of pT3N0M0. The prostate displayed three microfoci of intraparenchymatous Gleason 6 (3 + 3) adenocarcinoma, with no tumoral implication of the seminal vesicles. The surgical specimen presented negative surgical margins and negative distal ureteral margins.

**Figure 1.**
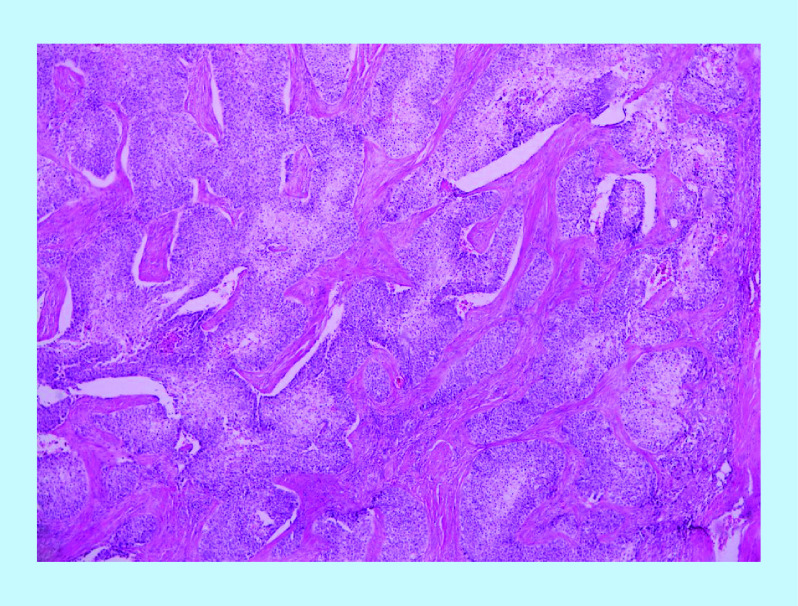
Pelvic computed tomography showing poorly limited hypodense lesions of corpora cavernosa.

**Figure 2.**
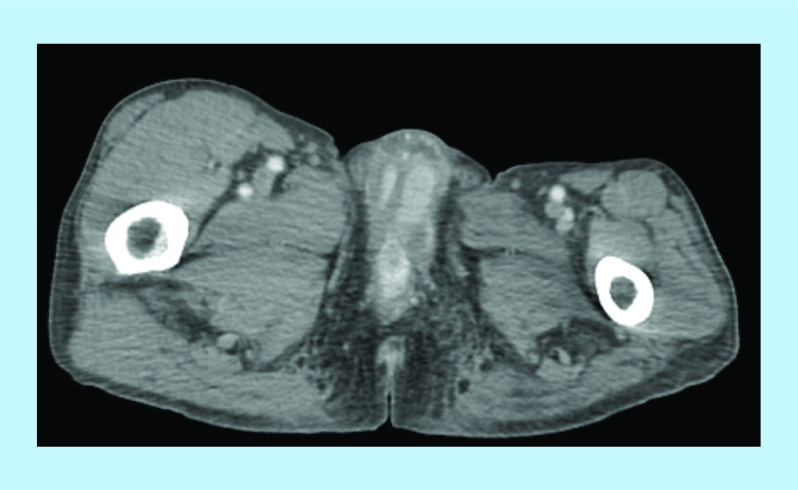
Pelvic computed tomography showing poorly limited hypodense lesions of corpora cavernosa.

The patient consulted 5 months after his surgery for a subacute onset of a painful penile induration and reported episodes compatible with stuttering priapism. He had a good performance status. Physical examination revealed a semi-erect penis and a considerable penile induration in regard of the corpora cavernosa. CT imaging of abdomen and pelvis identified isolated nonspecific, poorly limited, hypodense lesions of corpora cavernosa (Figures 1 & 2). Incisional biopsy of the palpated lesions revealed a poorly differentiated carcinoma with poor evaluation of surgical margins due to fragmentation of tissue specimen. No further imaging was performed due to financial impeachments.

**Figure 3.**
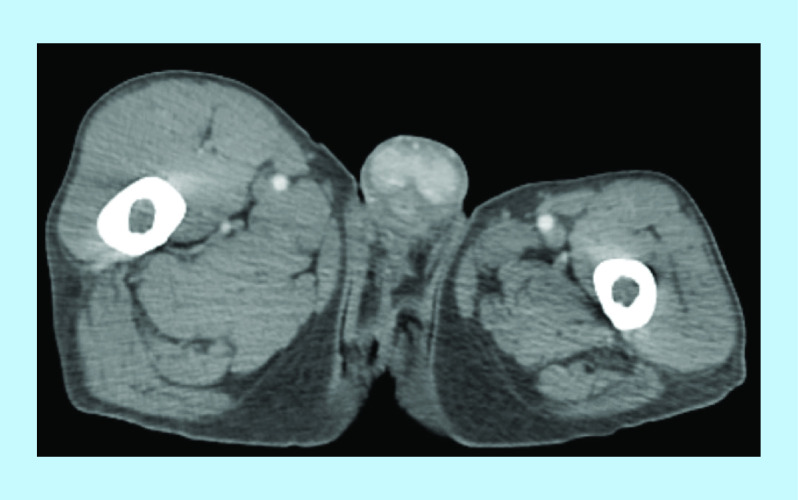
Hematoxylin and eosin stain showing vascular invasion of corpuscavernosus by urothelial carcinoma.

**Figure 4.**
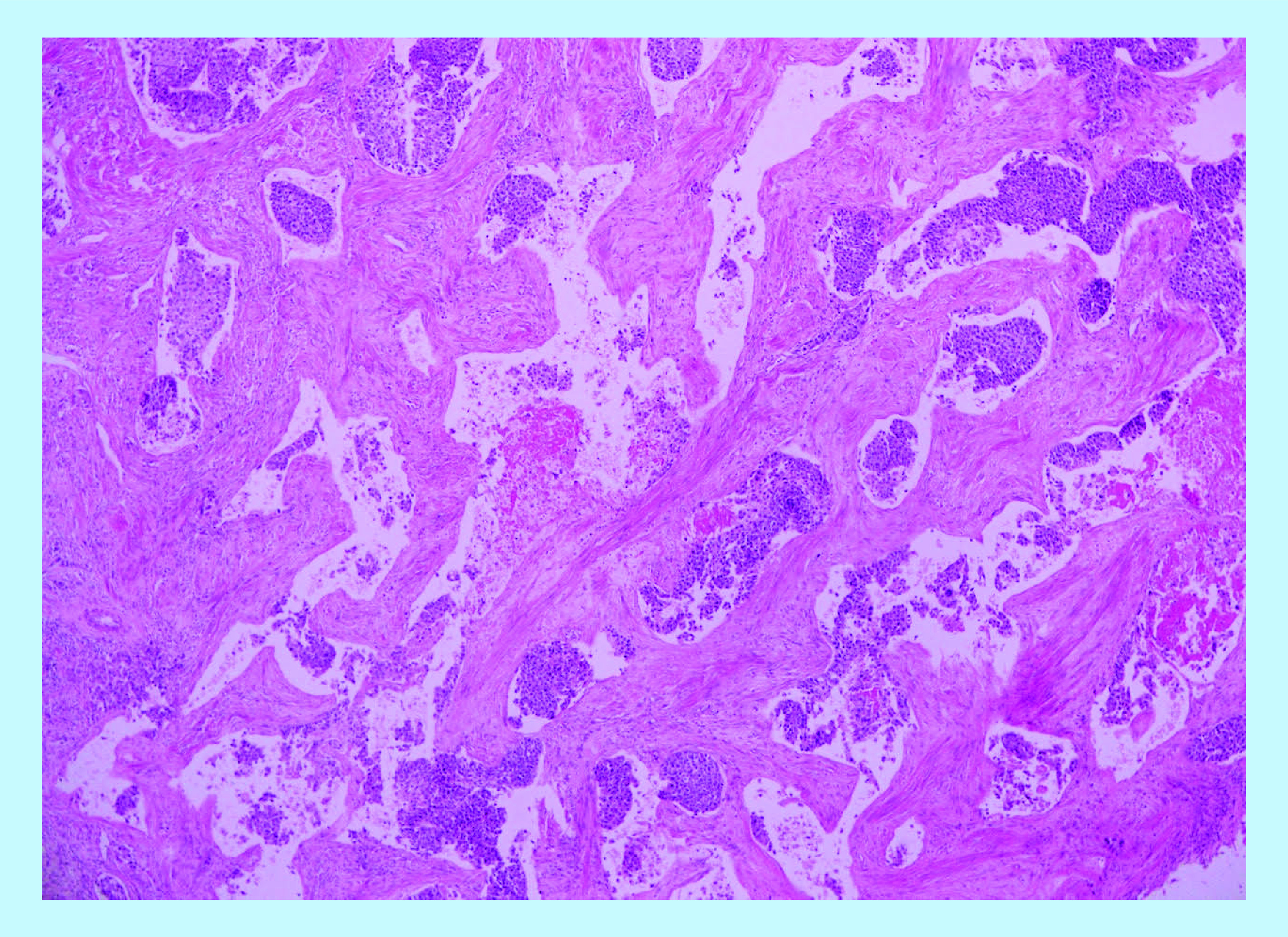
Hematoxylin and eosin stain showing massivevascular invasion of corpus cavernosus by urothelial carcinoma.

A total penectomy was performed, which revealed a bulky invasion of the corpora cavernosa by a UC, with a secondary extension to the corpus spongiosum and a focal involvement of the urethra. Tumoral vascular emboli were present within the specimen (Figures 3 & 4). Surgical margins were negative, including the remaining proximal urethra.

## Background

Pathological stage of the primary urothelial bladder tumor and nodal status are predictive factors of recurrence. In the majority of cases, recurrence occurs as distant metastases while local recurrences concern approximately 30% of cases [1]. Autopsy studies suggest that almost every organ could potentially be affected by metastases from urothelial bladder cancer. In 95% of cases, metastases concern the lymph nodes, lungs, liver and bones. On the other hand, penile recurrences are extremely rare according to their incidence in the medical literature [2–13].

Overall, approximately 370 cases of penile metastatic involvement have been reported in almost two centuries [14]. Penile metastatic disease is relatively rare, with the majority of primary cancers being urogenital, mainly prostate and bladder malignancies, which account for 34 and 30% of penile metastasis cases, respectively [7,15]. Penile metastases indicate a disseminated disease and therefore a poor prognosis [14]. Proposed mechanisms of penile infiltration by UC involve a retrograde venous route, a retrograde lymphatic route, an arterial spread, a direct extension, tumoral implantation and a iatrogenic pathway secondary seeding [16]. Most frequently, penile metastasis presents as penile nodules (51%) and/or priapism (27%). Patients may also consult for hematuria, urethral discharge, lower urinary tract symptoms [15,17] or even a cutaneous or Pagetoid lesion to the penis [18]. The occurrence of priapism, especially in the setting of nonurogenital primary tumors, is regarded as a stigmata of poor prognosis [15]. The vast majority of secondary penile involvement by a bladder UC have a metachronous pattern and occur concurrently with the development of other systemic metastases [15,19]. Precisely, two-thirds occur after a mean time of 18 months from diagnosis of the primary bladder tumor, whereas the remaining third displays a synchronous pattern with the primary bladder tumor [7]. Nonetheless, penile metastasis may present earlier or even many years after radical surgical treatment, as cases of recurrences have been reported as early as 6 months after cystectomy or as late as 2.5 years [10,20,21].

Metachronous penile metastasis of UC tend to occur in a setting of advanced-stage bladder cancer. The majority of reported cases mentioned a locally advanced disease with extravesical (pT3 according to TNM classification) extension of tumor, after radical cystoprostatectomy [7,8].

As for the therapeutic options, hormonal therapy, radiation therapy and chemotherapy have yielded poor results. Brachytherapy was proposed to assure local disease control for up to 1 year. Partial or local excision of penile recurrences globally yielded poor prognostic results as well, but patients with small isolated lesions seemed to have a better outcome when wide resection or total penectomy was performed [21,22].

In the setting of disseminated disease, the majority of patients with penile metastasis succumb with a year of cancer recurrence localized to the penis [23].

## Discussion

In the presented case, the patient consulted 7 months after the diagnosis of his muscle invasive bladder tumor, with a palpable penile nodule and priapism due to a unique metastasis located to the penis. The patient had undergone radical cystoprostatectomy 5 months prior. On definitive pathology from the cystoprostatectomy specimen, he was diagnosed with a locally advanced tumor extending to the perivesical fat with negative surgical margins (pT3a according to TNM classification). The urothelial infiltration of corpora cavernosa was considered to be a penile recurrence of his transitional cell bladder carcinoma rather than a *de novo* urethral primary carcinoma due to the normal findings of urethroscopy performed 5 months before his radical surgery. Furthermore, pathological findings on the total penectomy specimen demonstrated a focal-only infiltration of the urethra from a bulky urothelial recurrence within the corpora cavernosa.

To our knowledge, this case is one of the very few cases reported in the medical literature where a unique metastasis, located to the penis, occurs as early as 7 months after diagnosis of muscle-invasive urothelial bladder cancer. Imaging workup failed to demonstrate other distant recurrences or a disseminated disease as it is custom in penile metastasis of UC. Many case series report early UC recurrences located exclusively to the penis after radical surgical therapy but, failed to demonstrate the absence of other distant metastases.

The fact that the patient presented with an early unique metastasis located to the penis and that penectomy was performed resulting with negative surgical margins, may warrant a better prognosis for the patient, allowing them to avoid unnecessary chemotherapy or radiation therapy.

Since penile metastases almost always occur in the setting of disseminated disease, close follow-up of the patient is mandatory through tomographic and metabolic imaging in order to diagnose other distant recurrences of UC.

## Conclusion & future perspective

In conclusion, the following case highlights the importance of close follow-up of patients who underwent radical cystectomy for a muscle-invasive urothelial bladder cancer. Follow-up must include a thorough total-body physical examination searching for atypical lymphadenopathies, skin lesions, priapism, new-onset lower urinary tract symptoms or hematuria. Follow-up must be tailored to each patient according to general status and pathological staging of disease. Recurrences may present as early as few months postoperatively.

Penile metastases have only been mentioned in sparse case reports or in retrospective case series without separation or stratification of outcomes according to the primary tumor. Thus, due to underpowered studies and the low incidence of secondary penile recurrences, treatment recommendations tailored to this particular metastatic location are lacking, meaning additional research output must be put in that direction.

Additionally, in the setting of early metastatic recurrence from bladder UC, occurring within few months after radical surgery with preoperative imaging failing to detect any metastases, emerges the question of sensitivity of currently recommended imaging modalities in detecting potentially relevant metastatic seeding from bladder UC.

**Executive summary**

**Case presentation**

- A 77-year-old male, previously smoker, underwent a radical cystoprostatectomy with bilateral extended pelvic lymph node dissection for muscle-invasive bladder cancer. On previously performed diagnostic urethrocystoscopy, no malignant lesion was seen in the urethra.
- Preoperative thoracic, abdominal and pelvic computed tomography (CT) and magnetic resonance imaging demonstrated no signs of suspicious nodal involvement nor distant metastatic spread.
- Definitive pathology reported a urothelial pT3N0M0 bladder tumor. No urethral neoplastic involvement.
- The patient consulted 5 months after his surgery for a painful penile induration and stuttering priapism.
- CT imaging of abdomen and pelvis identified isolated nonspecific, poorly limited, hypodense lesions of corpora cavernosa.
- Total penectomy was performed and revealed a urothelial carcinoma infiltrating the corpora cavernosa, the corpus spongiosum and the urethra focally with presence of tumoral vascular emboli.

**Background**

- Penile recurrences of urothelial bladder cancer are pointed to as extremely rare according to their incidence in the medical literature.
- Penile metastases indicate a disseminated disease and therefore a poor prognosis.
- Most frequently, penile metastasis presents as penile nodules (51%) and/or priapism (27%).
- The occurrence of priapism, especially in the setting of nonurogenital primary tumors, is regarded as a stigmata of poor prognosis.
- Two-thirds of penile recurrences occur after a mean time of 18 months from diagnosis of the primary bladder tumor.
- The majority of reported cases mentioned a locally advanced disease with extravesical extension of tumor.
- Patients with small isolated lesions seemed to have a better outcome when wide resection or total penectomy was performed.

**Discussion**

- This case is one of the very few reported cases where a unique penile metastasis, occurs as early as 7 months after diagnosis of muscle-invasive urothelial bladder cancer.
- Penectomy was performed in negative surgical margins, which may warrant better prognosis for the patient and avoid unnecessary chemotherapy or radiation therapy.

**Conclusion & future perspective**

- Follow-up must be tailored to each patient according to general status and pathological staging of disease and must include a thorough physical examination.
- Additional research output must be put in the direction of implementing therapeutic recommendations for this rare entity.
